# Biomimetic Mechanism Transfer in Interior Environmental Comfort: A Systematic Mapping and Evidence-Stratified Framework

**DOI:** 10.3390/biomimetics11040225

**Published:** 2026-03-25

**Authors:** Dilek Yasar

**Affiliations:** Department of Interior Architecture, Istanbul Aydin University, Istanbul 34295, Türkiye; dilekyasar@aydin.edu.tr

**Keywords:** biomimetic mechanism transfer, indoor environmental quality, performance-based evaluation, evidence-stratified analysis, systematic mapping review

## Abstract

Biomimetic strategies have increasingly informed adaptive environmental systems; however, biomimetic mechanism transfer into interior environmental comfort remains unevenly operationalized and weakly evidence-stratified. Despite rapid post-2020 expansion of nature-inspired strategies, cross-domain translation across thermal comfort, indoor air quality (IAQ), visual comfort, and acoustic performance remains fragmented. This study addresses this gap by systematically mapping biomimetic mechanism transfer pathways within interior environmental systems, using biophilic strategies as a comparative baseline. A systematic mapping review was conducted following PRISMA 2020 guidelines to examine biomimetic mechanism transfer across interior environmental comfort domains. Studies were coded according to comfort domain, intervention scale, evidence type, and empirical strength. Results indicate three recurrent imbalances in the screened corpus: biophilic strategies dominate the literature (71.8%), intervention activity is concentrated at system scale and within multi-domain configurations, and acoustic bio-inspired optimisation is absent as a primary research domain. At the same time, the evidence base remains weakly stratified: only 10.3% of studies report statistically validated empirical findings, whereas 50.0% remain review-based or concept-led. To address these imbalances, the study proposes the Biomimetic Mechanism Transfer Mapping Framework (CPMF), a six-layer model linking biological logic, physical process activation, measurable IEQ outputs, empirical robustness, and implementation feasibility. The framework advances biomimetics by structuring mechanism translation into operational interior environmental performance systems.

## 1. Introduction

Biomimetic design has become a central discourse in contemporary sustainable architecture and environmental systems. In recent years, biomimicry and biophilic design have emerged as two distinct approaches to integrating biological principles within the built environment. While both draw inspiration from living systems, they diverge fundamentally in their operational logic. Biophilic design predominantly emphasises human–nature interaction, experiential restoration, and spatial–material qualities [1,2], whereas biomimetic strategies are typically oriented toward the translation of biological mechanisms into thermodynamic and environmental performance systems [3,4].

Post-2020 scholarship demonstrates rapid expansion of biophilic strategies across interior contexts, particularly in relation to daylight modulation, vegetation integration, and spatial configuration [5,6,7]. However, empirical validation of cross-domain environmental performance remains uneven. Visual comfort and perceived well-being are frequently foregrounded, while systematic integration of thermal, acoustic, and indoor air quality (IAQ) parameters is less consistently operationalized [8,9].

By contrast, biomimetic research has advanced primarily through adaptive façade systems, kinetic shading devices, and climate-responsive envelope optimisation [10,11,12]. Simulation-based modelling remains a predominant mode of evaluation in this domain, indicating a high degree of technical sophistication [13,14]. Yet computational evaluation frequently precedes longitudinal interior-scale validation, reflecting a persistent performance gap between modelling predictions and realised environmental outcomes [15].

Simultaneously, indoor environmental quality (IEQ) research increasingly advocates holistic integration of thermal comfort, visual performance, acoustic quality, and air quality within unified assessment frameworks [16,17,18]. Multi-parameter optimisation models and energy–IEQ integration studies underscore the need for cross-domain environmental synthesis [19,20]. However, methodological heterogeneity persists across weighting systems, indicator definitions, and validation protocols [16,17,18,19,20,21]. Acoustic and IAQ parameters, in particular, remain inconsistently embedded within integrated frameworks [22,23]. Taken together, three structural asymmetries emerge across biomimetic and IEQ scholarship:

- Conceptual and experiential expansion without systematic cross-domain operationalization in biophilic research.
- Simulation-dominant thermodynamic optimisation in biomimetic research with uneven empirical deployment.
- Persistent fragmentation in IEQ integration, particularly across acoustic and IAQ domains.

Despite the proliferation of biologically inspired discourse, no structured analytical framework currently formalises biomimetic mechanism transfer from biological logic to measurable indoor comfort outcomes, empirical robustness, and implementation feasibility within a unified comparative structure. Existing studies tend to assess biophilic and biomimetic approaches independently, without systematic cross-domain mapping or differentiation by evidence strength.

To address this gap, the study adopts indoor environmental quality (IEQ) as its central evaluative lens. IEQ offers the most operationally coherent basis for assessing interior environmental performance because it consolidates thermal comfort, visual comfort, acoustic quality, and indoor air quality within a common assessment logic. Since biomimetic design often oscillates between biological metaphor and technical application, a structured evaluative lens is required to determine whether transferred mechanisms produce measurable effects in actual indoor settings. IEQ is therefore used here not as a generic sustainability indicator, but as the analytical platform that enables cross-domain comparison, evidence stratification, and performance-oriented interpretation of biomimetic interior strategies.

Building on this lens, the study advances biomimetics by systematising the translation of biological mechanisms into interior environmental performance systems. Rather than focusing only on adaptive façade typologies or envelope optimisation, it formalises biomimetic transfer across measurable indoor comfort domains while integrating empirical validation and implementation feasibility into a single comparative structure.

On this basis, all four principal IEQ domains—thermal comfort, visual comfort, acoustic comfort, and indoor air quality—are included because a multi-domain evaluation of indoor environmental conditions remains incomplete if any major comfort dimension is excluded. Each domain affects user experience and interior performance through distinct yet interrelated mechanisms: thermal conditions shape physiological comfort; visual conditions influence glare control, circadian support, task performance, and perceptual legibility; acoustic conditions affect communication quality, cognitive performance, behavioural tolerance, and psychological well-being; and indoor air quality influences respiratory health, alertness, and environmental acceptability. Considering these domains together is essential not only for analytical completeness but also for testing the study’s central argument that biomimetic mechanism transfer should be assessed through measurable outputs across the full spectrum of indoor comfort. The reviewed literature also reveals a marked asymmetry across domains: visual and thermal bio-inspired strategies are considerably more developed, whereas acoustic and IAQ-oriented mechanisms remain limited. The near absence of acoustic bio-inspired optimisation, in particular, signals both an IEQ integration problem and a broader biomimetic research gap. This imbalance strengthens the need for a comparative and evidence-aware framework.

Accordingly, the present study addresses this gap through a systematic mapping review of post-2020 literature indexed in Scopus and Web of Science, focusing on four principal indoor comfort domains: thermal comfort, visual comfort, IAQ, and acoustic comfort. By comparatively analysing biomimetic interior strategies and using biophilic approaches as a structured baseline, the study proposes the Biomimetic Mechanism Transfer Mapping Framework (CPMF).

The CPMF repositions biomimetic interior design from conceptual analogy toward operational environmental performance logic. In doing so, it introduces an implementation-constraint layer that extends performance evaluation into translational feasibility, thereby contributing a structured, mechanistic, and evidence-aware model to contemporary biomimetics research.

## 2. Literature Review

### 2.1. Biophilic Interior Research: Expansion Without Structural Integration

Post-2020 scholarship demonstrates a rapid expansion of biophilic design within interior and building contexts. Recent reviews and empirical studies highlight increased attention to natural light modulation, material tactility, vegetation integration, and spatial configuration as contributors to indoor environmental quality (IEQ) and occupant well-being [1,2,5]. Similarly, Tabassum [6] proposes evaluation frameworks linking biophilic principles to building assessment systems, while Thampanichwat [7] emphasises the architectural language and conceptual evolution of biophilic integration. 

However, despite thematic coherence, methodological dispersion remains evident. McGee and Park [8] note the predominance of visual–material emphasis within interior-focused biophilic discourse, often privileging perceptual and experiential dimensions over measurable environmental activation. While biophilic design is frequently associated with improved IEQ outcomes [2], systematic cross-domain performance validation—particularly across thermal, acoustic, and IAQ parameters—remains uneven.

Thus, contemporary biophilic research exhibits strong conceptual growth but limited structural integration across measurable environmental performance domains.

### 2.2. Biomimetic Mechanism Transfer in Adaptive Environmental Systems

Contemporary biomimetic research increasingly seeks to translate biological mechanisms into thermodynamic, daylight, and airflow regulation processes within architectural systems. In parallel, biomimetic architectural research has largely advanced through adaptive façade systems, climate-responsive skins, and bio-inspired envelope optimisation [3,4]. Computational modelling plays a dominant role in evaluating thermodynamic efficiency and environmental modulation [10,11,12]. Biomimetic envelope systems increasingly incorporate biologically inspired dynamic control logics, including hygro-responsive and other adaptive actuation mechanisms [13,14]. More recent studies extend this trajectory through flower-inspired kinetic façade concepts, climate-responsive shading systems, and adaptive façade–glazing integration strategies designed to improve daylight performance and glare control [24,25,26].

While such studies demonstrate high modelling sophistication, empirical validation at interior scale is comparatively limited. Hosseini [27] and Alyahya et al. [28] illustrate adaptive façade optimisation through simulation-based daylight and thermal performance metrics; however, post-occupancy measurement and multi-domain IEQ integration remain less common. Critical reflections on biomimetic design methodology further underscore conceptual ambiguities and translation gaps between biological analogy and architectural implementation [29]. Consequently, biomimetic research appears technically advanced at the modelling level but structurally uneven in validated interior-scale deployment.

### 2.3. Indoor Environmental Quality (IEQ) Integration and Model Heterogeneity

Concurrently, IEQ research increasingly advocates holistic integration of thermal comfort, visual performance, acoustic quality, and air quality within unified evaluation frameworks [16,17]. Studies have proposed multi-parameter optimisation models for office and institutional environments [18,19,20,21,22,23,27,28,29,30], as well as combined energy–IEQ assessment strategies [19,20].

Nevertheless, persistent methodological heterogeneity remains. Roumi [16] demonstrate variability in IEQ weighting systems and indicator definitions, while Zhang [17] identify inconsistencies in model operationalisation across studies. Field measurement research [21,22,23,27,28,29,30,31] confirms the difficulty of simultaneously optimising all four IEQ domains in applied settings.

Importantly, IEQ frameworks frequently privilege thermal and visual parameters, while acoustic and IAQ integration remains less systematically embedded [9,10,11,12,13,14,15,16,17,18,19,20,21,22]. This structural imbalance mirrors patterns observed in bio-inspired interior research.

### 2.4. Performance Validation, Simulation Dominance, and the Evidence Gap

A growing body of research highlights the divergence between computational modelling and real-world performance outcomes. Donn [17] characterises this divergence as a “performance gap,” particularly where simulation-based evaluation precedes empirical verification. Brzezicki [32] similarly demonstrates the dominance of conceptual and modelling approaches in adaptive shading literature, often without longitudinal performance validation.

Review-based assessments in this area also emphasise the need for greater empirical robustness and for bridging digital models with larger-scale experimental and in-field testing [13]. Meanwhile, design-led sustainability innovation research acknowledges that performance claims must be supported by measurable environmental metrics rather than conceptual alignment alone [33]. Collectively, these studies reveal three structural patterns across biomimetic and IEQ scholarship:

- Strong emphasis on modelling and simulation.
- Fragmented integration across environmental comfort domains.
- Limited stratification of empirical validation levels.

Despite the rapid expansion of biomimetic and biologically informed design discourse, no unified analytical model currently links design strategy, physical activation mechanisms, measurable comfort outputs, empirical robustness, and implementation feasibility within a structured comparative framework.

### 2.5. Research Gap

Across biophilic interior research, biomimetic envelope optimisation, and IEQ integration literature, the absence of systematic cross-domain comparison and evidence-tier differentiation remains evident. Acoustic bio-inspired optimisation is particularly underrepresented, while implementation feasibility is rarely embedded as an explicit evaluative layer.

Accordingly, there remains a need for a structured, mechanistic, and performance-oriented analytical framework capable of mapping bio-inspired design strategies across comfort domains while simultaneously integrating empirical validation strength and translational constraints.

The present study addresses this gap through the development of the Biomimetic Mechanism Transfer Mapping Framework (CPMF), grounded in systematic mapping of post-2020 literature.

The novelty of the present study lies in how it differs from earlier strands of research. Previous biophilic studies have predominantly concentrated on perceptual restoration, visual exposure, and spatial–material experience, often without systematic cross-domain validation across measurable indoor performance parameters. Biomimetic studies, by contrast, have largely advanced through adaptive façades, kinetic envelopes, and simulation-driven thermodynamic optimisation, but have less frequently compared performance intersections across interior comfort domains or differentiated findings according to empirical robustness. IEQ studies have provided important integrative assessment models, yet they have generally not formalised biomimetic mechanism transfer as a structured analytical pathway linking biological logic, performance output, evidence strength, and implementation feasibility. In this respect, the originality of the present study lies not simply in reviewing the recent literature, but in comparatively reorganising previously disconnected bodies of work within a single evidence-stratified and cross-domain framework.

## 3. Methodology

### 3.1. Research Design

This study adopts a systematic mapping review design structured in accordance with the PRISMA 2020 reporting guidelines. The aim is not merely to synthesise existing literature, but to systematically examine biomimetic mechanism transfer within interior environmental comfort systems, using biophilic strategies as a structured comparative baseline.

Unlike conventional narrative reviews, this study applies a structured, reproducible screening and coding procedure to map the distribution of biomimetic and biologically informed design strategies across defined performance domains. The review focuses exclusively on building- and interior-scale applications and examines four principal indoor environmental comfort domains:

- Thermal comfort;
- Indoor air quality (IAQ);
- Visual comfort;
- Acoustic comfort.

The methodological framework integrates multi-database searching, transparent duplicate removal, explicit inclusion–exclusion criteria, and structured coding procedures to ensure analytical rigour and reproducibility.

### 3.2. Data Sources and Search Strategy

Two major scientific databases were selected to ensure broad disciplinary coverage:

- Scopus;
- Web of Science Core Collection;

The search was conducted in January 2026. Database-specific search syntax was applied; however, the conceptual query structure remained consistent across platforms. The core search string was as follows:

(“biophilic design” OR biophilia OR biomimicry OR “biomimetic design”)

AND

(“thermal comfort” OR “acoustic comfort” OR “indoor air quality” OR “visual comfort” OR lighting)

AND

(“interior design” OR “building design” OR “built environment”)

The publication period was limited to 2020–2025 to capture contemporary developments in performance-driven sustainable interior strategies.

Searches were restricted to Title–Abstract–Keywords fields in Scopus and Topic fields in Web of Science to ensure conceptual precision while maintaining disciplinary breadth. Only peer-reviewed journal articles and review articles were included. Conference proceedings, book chapters, editorials, and non-peer-reviewed materials were excluded to ensure methodological robustness and comparability of findings.

This review follows the PRISMA 2020 reporting guidelines for systematic and scoping reviews. A completed PRISMA-ScR checklist is provided as supplementary material [34]. No review protocol was prospectively registered.

### 3.3. Identification, Duplicate Removal, and Study Selection

The initial database search yielded 53 records from Scopus and 47 records from Web of Science, resulting in 100 identified records.

All records were exported in full-record format including DOI metadata to enable precise duplicate detection. Duplicate removal was performed using DOI matching as the primary identifier. Twelve duplicate records were identified and removed, resulting in 88 unique records.

**Figure 1:**
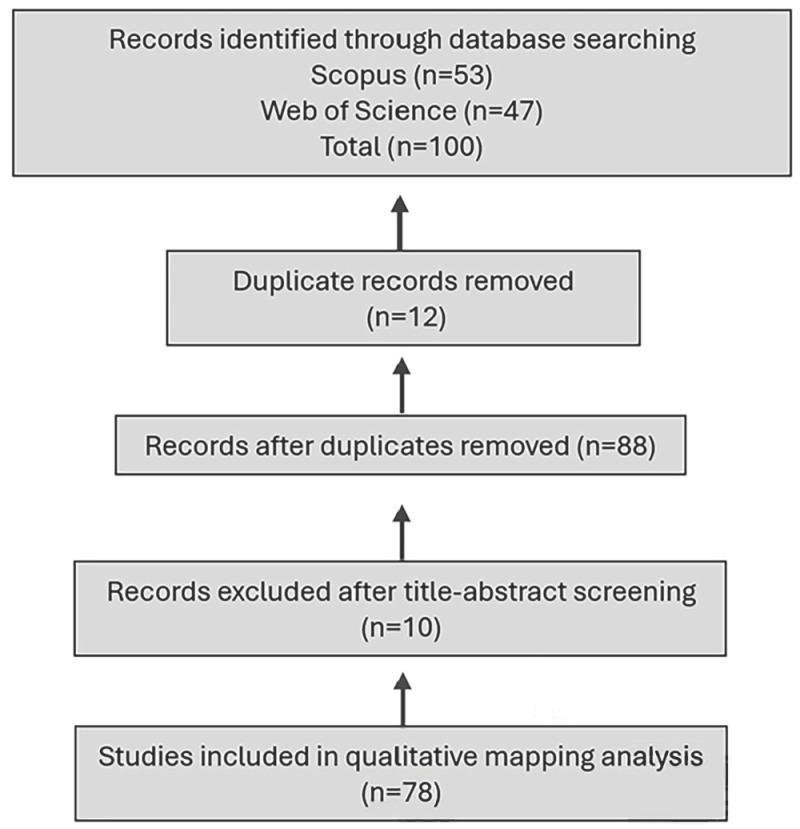
PRISMA 2020 flow diagram illustrating the study selection process.

Title–abstract screening was then conducted based on predefined eligibility criteria. Following screening, 10 records were excluded. A total of 78 studies met the inclusion criteria and were included in the final qualitative mapping analysis (Figure 1). The final number of included studies (*n* = 78) was not predetermined as a fixed sample size; rather, it emerged from the PRISMA-guided screening process as the set of records that satisfied the predefined eligibility criteria.

### 3.4. Screening and Eligibility Criteria

Title–abstract screening was conducted by the author using predefined inclusion and exclusion criteria to minimise conceptual drift and ensure procedural consistency.

Inclusion Criteria—Studies were included if they:

- Explicitly addressed biomimetic mechanism transfer, biophilic strategies (as comparative reference), or hybrid biologically informed design approaches.
- Operated at building or interior scale.
- Reported measurable indoor environmental performance outcomes or explicitly analysed performance-relevant indoor environmental parameters, mechanisms, or evaluation frameworks even when no original empirical or simulation-based outputs were generated.
- Addressed at least one of the four predefined comfort domains (thermal, IAQ, visual, or acoustic).

Exclusion Criteria—Studies were excluded if they:

- Focused solely on psychological perception without measurable environmental metrics (E2).
- Addressed urban-scale or outdoor landscape contexts without indoor environmental relevance (E1).
- Were purely conceptual without reference to measurable performance outcomes (E3).
- Addressed non-built-environment domains (e.g., robotics or automotive systems) (E5).

This structured screening ensured that the final dataset was performance-oriented rather than perception-only.

### 3.5. Data Extraction and Coding Procedure

A structured coding matrix was developed to enable systematic comparison across the included studies. Each study was coded according to design approach (biophilic, biomimetic, or hybrid), primary comfort domain (thermal, indoor air quality, visual, acoustic, or multi-domain), intervention type (e.g., façade system, vegetation system, HVAC strategy), evidence type (experimental, simulation, mixed, or review), reported performance metrics (e.g., PMV, CO_2_ concentration, illuminance, RT60), and evidence strength (A–D scale).

In this coding structure, “multi-domain” refers to studies that simultaneously addressed at least two indoor environmental comfort domains within the same analytical or performance-evaluation framework. The category therefore does not imply full integration of all four IEQ domains, but rather denotes cross-domain treatment beyond a single-domain focus.

Evidence strength was classified as follows:

A—Empirical measurement with statistical validation;

B—Empirical measurement without detailed statistical reporting;

C—Simulation-based performance evaluation;

D—Review-based or conceptual analysis.

For coding purposes, the distinction between simulated and conceptual evidence was made according to the presence or absence of original performance-generating analytical procedures. Level C was assigned only when a study produced quantified indoor environmental performance outputs through simulation-based modelling (e.g., daylight simulation, CFD, thermal/energy modelling, airflow modelling, or coupled digital performance analysis), even in the absence of physical measurement. By contrast, Level D was assigned to review-based, conceptual, taxonomic, or design-propositional studies that did not generate original performance outputs through either empirical measurement or simulation. Thus, simulated evidence was treated as analytically stronger than conceptual evidence because it produced model-derived performance values, whereas conceptual evidence remained interpretive, descriptive, or framework-oriented without direct performance generation. Importantly, Level D classification should not be interpreted as including all conceptual studies. During screening, studies were excluded only if they were purely discursive and lacked explicit relevance to indoor environmental performance. Review-based or conceptual studies were retained only when they addressed performance-relevant comfort domains, mechanisms, parameters, or evaluative frameworks, even if they did not generate original empirical measurements or simulation outputs. Thus, exclusion at screening and Level D classification served different methodological purposes: the former determined relevance to the review scope, whereas the latter captured evidential robustness within the included corpus.

To enhance methodological robustness, a subset of the dataset (15% of the included studies) was re-examined by the author after the initial coding phase to assess coding stability and categorical consistency. Ambiguous cases were revisited through iterative comparison with the predefined coding rules. This procedure improved the internal coherence of the categorical mapping framework and reduced interpretative drift across the dataset.

The coding procedure enabled cross-domain comparison and systematic identification of underrepresented performance intersections across biomimetic and biophilic interior strategies. Intervention scale classification revealed that 41% of studies operated at the system scale (envelope or HVAC-integrated strategies), 28% at the component scale (façade modules, shading devices), 19% at the material scale (bio-based or porous materials), and 12% at the spatial configuration level. As shown in Table 1, system-scale interventions were the most frequent category in the screened corpus. This indicates that the reviewed literature, at the aggregate dataset level, is more strongly oriented toward technically integrated environmental strategies than toward spatially configured interventions. However, because intervention scale was summarised distributionally rather than retained as a record-level variable cross-tabulated by design approach, Table 1 should be interpreted as a descriptive corpus profile rather than as direct evidence that biophilic or biomimetic approaches are systematically associated with particular scales.

Accordingly, scale-related observations are used here as descriptive context for the subsequent cross-domain analysis rather than as independently demonstrative evidence of approach-specific divergence.

### 3.6. Analytical Strategy

The analytical process proceeded in three stages:

- Quantitative mapping of biomimetic mechanism transfer across interior comfort domains.
- Cross-tabulation of intervention type and evidence type.
- Cross-tabulation of intervention type and evidence strength (A–D).
- Identification of underrepresented bio-inspired performance intersections.

Because intervention scale was summarised distributionally at corpus level rather than retained as a separate record-level cross-tabulation field in the final analytical spreadsheet, a formal intervention scale × evidence strength contingency analysis could not be performed in the present study. Accordingly, any comments on robustness patterns across scales should be interpreted as descriptive rather than statistically demonstrated. This structured mapping formed the empirical foundation for the development of the Biomimetic Mechanism Transfer Mapping Framework (CPMF), presented in Section 4.6.

In addition to categorical coding, the extraction framework was structured along a causal-operational logic chain linking design strategy to measurable environmental outcomes. Each study was interpreted through a six-step analytical pathway: (1) design strategy, (2) underlying bio-logic mechanism, (3) activated physical process, (4) comfort output parameter, (5) evidence strength classification, and (6) reported or inferred implementation constraints (e.g., maintenance demand, cost intensity, regulatory compatibility). In the present study, physical process activation refers to the specific biophysical operation through which a design strategy produces an indoor environmental effect, such as heat-gain modulation, airflow regulation, moisture buffering, pollutant adsorption, daylight redistribution, or sound attenuation. Implementation feasibility refers to the practical deployability of that strategy under interior application conditions, including maintenance burden, cost intensity, durability, control complexity, code compliance, and integration with existing building systems. This layered analytical structure enabled not only distribution mapping but mechanistic comparison across bio-inspired performance strategies.

This analytical structure explicitly operationalises biomimetic mechanism transfer, enabling structured comparison between biological analogues and measurable indoor environmental performance outcomes.

### 3.7. Supplementary Bibliometric Overview

To provide an overall view of the final screened corpus, a supplementary bibliometric overview was conducted on the included studies retrieved from Scopus and Web of Science (*n* = 78). This descriptive step comprised publication-year distribution and author keyword co-occurrence mapping based on the final screened dataset. Author keywords were exported from the final screened records, manually cleaned to merge obvious spelling, singular–plural, and hyphenation variants, and then visualised for descriptive overview only. Because this step was supplementary rather than inferential, no threshold-based cluster optimisation or network statistics were used in the interpretation. It was used as a complementary overview only; the primary methodological design of the study remained a PRISMA-guided systematic mapping review based on structured screening, coding, and evidence-stratified comparative analysis.

## 4. Results and Framework Development

### 4.1. Descriptive Bibliometric Overview of the Final Screened Corpus

**Figure 2:**
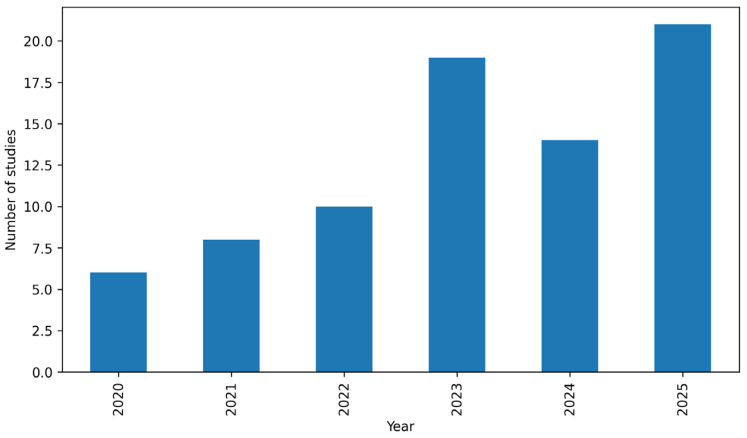
Publication year distribution of the final screened Scopus/WoS corpus (*n* = 78).

As a descriptive bibliometric overview of the final screened corpus, the publication-year distribution (Figure 2) indicates a marked increase in relevant studies in the later part of the review window, confirming the recent acceleration of interest in biomimetic and biophilic approaches to interior environmental performance. The author keyword co-occurrence structure further shows a strong thematic concentration around biophilic design, biomimicry, indoor environmental quality, biophilia, and thermal/visual–environmental concerns. Together, these descriptive patterns support the systematic mapping results by confirming that the literature clusters around visually and environmentally oriented strategies, while other domains remain comparatively less developed.

**Figure 3:**
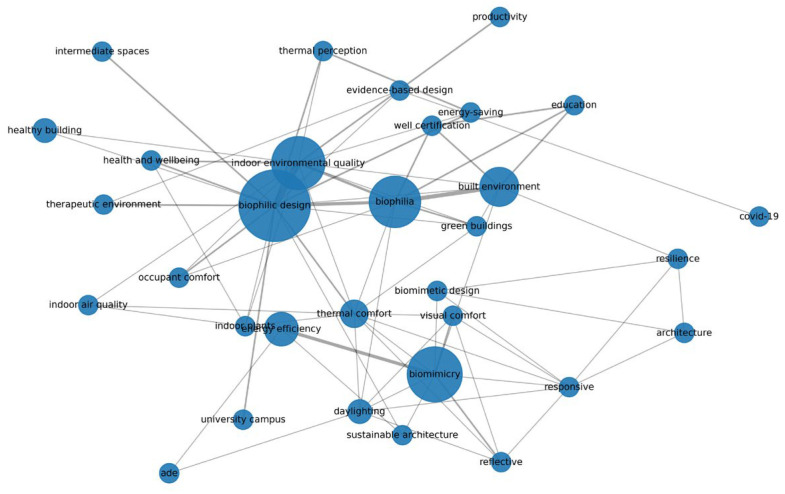
Author Author keyword co-occurrence network of the final screened Scopus/WoS corpus (*n* = 78). Node size and line thickness indicate keyword frequency and link strength, respectively.

Figure 3 illustrates the thematic clustering of the reviewed corpus, with biophilic design, biomimicry, indoor environmental quality, and related performance concepts forming the most visible keyword structure.

### 4.2. Cross-Domain Performance Mapping

To move beyond simple frequency analysis and toward explicit evaluation of biomimetic mechanism transfer across interior environmental systems, the analysis begins with the cross-tabulation of design approach and primary indoor comfort domain (see Table 2).

As presented in Table 2, biomimetic and biophilic approaches are unevenly distributed across thermal, IAQ, visual, acoustic, and multi-domain configurations. Three analytically significant patterns emerge.

First, biophilic strategies are strongly concentrated in multi-domain applications (67.9%), followed by visual comfort (26.8%), while thermal applications remain limited (5.4%). This indicates that biophilic research frequently operates at the level of spatial configuration and experiential integration rather than targeted thermal optimisation.

Second, biomimetic strategies demonstrate comparatively stronger thermal orientation (26.3%) and are predominantly clustered within multi-domain interventions (52.6%). When read alongside the overall scale distribution in Table 1, this pattern is consistent with—rather than independently demonstrative of—the broader system-scale orientation of the dataset.

Third, acoustic performance is entirely absent as a primary domain across all approaches.

Taken together, Table 2 reveals that bio-inspired interior research is not evenly distributed across environmental performance categories but instead clusters around specific domain–approach pairings. The absence of acoustic bio-inspired optimisation constitutes a significant research gap, particularly given the increasing relevance of soundscape quality in contemporary interior environments.

### 4.3. Domain-Level Imbalance and Environmental Prioritisation

To further clarify environmental prioritisation patterns independent of design approach, the overall allocation of studies across primary comfort domains is presented in Table 3 (*n* = 78).

As shown in Table 3, multi-domain studies account for 62.8% of the dataset, followed by visual comfort (25.6%) and thermal comfort (10.3%). IAQ appears as a primary focus in only 1.3% of studies, while acoustic comfort remains entirely unrepresented (0%).

Although the prevalence of multi-domain studies may initially suggest integrative environmental modelling, closer examination indicates that many combine visual and thermal parameters rather than comprehensively addressing all four comfort domains. Thus, the dominance of the multi-domain category should be interpreted as evidence of partial cross-domain integration rather than fully holistic IEQ coverage.

When Table 2 and Table 3 are interpreted collectively, a set of structural asymmetries becomes evident:

- Visual dominance within biophilic discourse;
- Thermal concentration within biomimetic strategies;
- Minimal IAQ-exclusive modelling;
- A near-total absence of acoustic bio-inspired performance research.

These findings indicate that contemporary biomimetic interior research remains selectively integrated rather than holistically environmental.

### 4.4. Overall Approach Distribution and Paradigm Imbalance

The aggregate distribution of design approaches is summarised in Table 4. Biophilic strategies constitute 71.8% of the included dataset, while biomimetic strategies account for 24.4% and hybrid strategies 3.8%.

This imbalance indicates that biomimetic mechanism transfer remains comparatively underdeveloped within interior environmental systems. Biomimetic strategies—despite their technical potential for environmental optimisation—remain comparatively underrepresented. Importantly, when Table 2, Table 3 and Table 4 are interpreted collectively, a disciplinary bifurcation becomes apparent:

- Biophilic research tends to prioritise experiential, visually oriented environmental configurations.
- Biomimetic research tends to prioritise thermal-envelope and system-based performance mechanisms.

This structural division reinforces the need for a unified evaluative structure capable of bridging experiential and biophysical performance logics.

### 4.5. Empirical Robustness and Validation Gaps

Beyond domain distribution, methodological maturity varies significantly. As presented in Table 5 (*n* = 78), review-based studies constitute 26.9% of the dataset, while experimental (23.1%) and simulation-based (17.9%) studies collectively represent 41.0%. Mixed-method studies account for 9.0%, and 23.1% of studies exhibit limited methodological specification.

Further differentiation is provided in Table 6. Evidence Strength Classification (*n* = 78). Only 10.3% of studies achieve Level A (empirical measurement with statistical validation), while 21.8% fall under Level B, 17.9% under Level C (simulation-based), and 50% remain review-based or conceptually structured (Level D).

Thus, although bio-inspired interior strategies are increasingly discussed, robust empirical validation remains uneven and fragmented.

This methodological imbalance strengthens the argument for a structured comparative model that integrates thematic orientation with empirical credibility.

While evidence type reflects methodological format, evidence strength captures empirical robustness; therefore, both dimensions must be interpreted jointly. The distribution of evidence strength levels across included studies is summarised in Table 6.

While evidence type reflects methodological format, evidence strength captures empirical robustness; therefore, both dimensions must be interpreted jointly. To operationalise this joint interpretation, we report the distribution of evidence strength across intervention types (Table 7), highlighting where empirically robust validation is concentrated versus predominantly conceptual evidence.

Although a formal comfort-domain × evidence-strength contingency table was not generated in the present dataset, the combined reading of Table 2, Table 3, Table 6 and Table 7 suggests that empirical robustness is unevenly distributed across bio-inspired performance clusters. Validation appears to be more concentrated in thermally and system-oriented intervention clusters, whereas visually oriented and biophilic studies are more frequently represented within concept-led or review-based literature. This pattern should be interpreted as indicative rather than inferentially confirmed and therefore warrants record-level cross-tabulation in future research.

### 4.6. Development of the Biomimetic Mechanism Transfer Mapping Framework (CPMF)

The structural asymmetries identified in Table 2, Table 3, Table 4, Table 5 and Table 6 form the empirical foundation for the proposed Biomimetic Mechanism Transfer Mapping Framework (CPMF) (see Table 8). Importantly, the CPMF was not conceptually imposed a priori but inductively derived from empirical clustering patterns observed across Table 2, Table 3, Table 4, Table 5 and Table 6. The framework therefore reflects data-driven structural relationships rather than theoretical abstraction alone. The CPMF should therefore be interpreted as an internally grounded analytical synthesis rather than as a fully externally validated predictive tool. Within the scope of the present study, its validity derives from three methodological anchors: first, its inductive derivation from recurrent distributional and cross-tabulated patterns identified across Table 2, Table 3, Table 4, Table 5 and Table 6; second, its dependence on explicitly defined coding dimensions linking mechanism, comfort output, evidence strength, and intervention scale; and third, the internal consistency control provided through author-led re-examination of a 15% subset of the dataset after the initial coding phase. At the same time, external validation through applied case testing, expert evaluation, or prospective project-based verification lies beyond the current scope and remains a necessary next step for future research.

The framework visualises biomimetic mechanism transfer pathways from biological logic to measurable indoor environmental comfort outcomes and feasibility constraints.

Unlike conventional taxonomic classifications, the CPMF is structured as a six-layer causal-operational chain linking design intent to measurable environmental performance and implementation feasibility. The framework consists of the following analytically connected layers:

- Design Strategy (biophilic, biomimetic, hybrid);
- Bio-logic Mechanism (e.g., evapotranspiration, adaptive shading, hygroscopic buffering, porous diffusion);
- Physical Process Activation (heat transfer modulation, airflow regulation, pollutant adsorption, daylight redistribution);
- Comfort Output Parameter (PMV/PPD, operative temperature, CO_2_/VOC concentration, illuminance, glare index, RT60);
- Evidence Strength (A–D tier classification based on empirical validation);
- Implementation Constraint Layer (maintenance intensity, cost implication, scalability, regulatory alignment, durability).

The inclusion of the implementation constraint layer extends the CPMF beyond performance mapping and introduces translational feasibility as a critical evaluative dimension. This allows differentiation between theoretically promising strategies and practically deployable solutions.

Operationally, Table 8 should be read as a condensed cross-domain summary of the CPMF, organising the screened literature through the intersection of biophysical mechanisms and comfort domains while also incorporating literature density, evidence strength, and implementation-related constraints as comparative interpretive layers.

By structuring biomimetic and biologically informed strategies along this mechanistic-performance-feasibility continuum, the CPMF transforms fragmented literature clusters into a systematic, comparative, and implementation-aware analytical model. This differentiates the present study from earlier reviews and conceptual syntheses in three respects. First, unlike prior biophilic research that has often privileged experiential and visually oriented benefits, the present framework compares thermal, visual, IAQ, acoustic, and multi-domain pathways within one structure. Second, unlike much of the biomimetic literature, which has tended to privilege simulation-led façade or envelope optimisation, the CPMF evaluates mechanism transfer in relation to empirical validation and interior-scale environmental outcomes. Third, unlike conventional IEQ integration studies, the framework incorporates implementation feasibility as an explicit evaluative layer, thereby extending performance assessment toward translational applicability. This study contributes to the field in five distinct ways:

- It provides, to the author’s knowledge, one of the first structured cross-domain comparisons of biophilic and biomimetic interior strategies within measurable indoor comfort frameworks.
- It quantifies domain-level asymmetries, explicitly identifying the absence of acoustic bio-inspired optimisation.
- It integrates empirical validation tiers into comfort-domain mapping, revealing methodological fragmentation.
- It introduces an implementation constraint layer, extending performance analysis into feasibility assessment.
- It proposes the Biomimetic Mechanism Transfer Mapping Framework (CPMF) as a replicable, mechanistic, and performance-operational analytical model.

These findings collectively indicate that the evolution of bio-inspired interior research is not merely a thematic expansion but a structurally differentiated performance discourse.

### 4.7. Illustrative Retroactive Application of the CPMF

To demonstrate that the CPMF functions as more than a static classification device, two representative studies from the screened corpus are retroactively interpreted through its six-layer structure (Table 9). This illustrative step is not intended as external validation in a predictive sense; rather, it serves to show how the framework can organise mechanism translation pathways from biological logic to measurable environmental outputs and implementation constraints.

**Table 9:** Illustrative retroactive application of the CPMF to two representative studies from the screened corpus.

Study	Design Strategy	Bio-Logic Mechanism	Physical Process Activation	Comfort Output Parameter	Evidence Strength	Implementation Constraint Layer
Alyahya et al. [28]	Biomimetic	Ventilated opaque façade inspired by biological thermoregulation logic	Airflow regulation and heat-gain modulation	Thermal performance/ventilation-related environmental control	C (simulation-based performance evaluation)	Climate-specific applicability, façade integration complexity, maintenance and operational feasibility
Farmani et al. [12]	Biomimetic	Kinetic façade inspired by Venus flytrap motion logic	Daylight redistribution and glare modulation through responsive façade movement	Illuminance/glare-related visual comfort metrics	C (simulation-based performance evaluation)	Actuation complexity, control reliability, cost, durability, and calibration requirements

These examples show that the CPMF does not merely label studies according to broad thematic categories. Rather, it structures each study as a mechanism–translation sequence linking biological analogy to physical process activation, measurable comfort output, evidential status, and translational constraints. In this way, the framework makes explicit where a study is strong—for example, in performance modelling—and where it remains limited, such as in empirical validation or implementation feasibility. This demonstrates the practical analytical utility of the CPMF within the scope of the present review.

## 5. Discussion

### 5.1. Repositioning Biomimetic Interior Design as Performance Logic

The findings indicate that biomimetic mechanism transfer within interior environmental systems remains structurally differentiated and unevenly validated across comfort domains. While biophilic design dominates the literature (71.8%), its application is primarily concentrated in visually oriented and multi-domain experiential configurations. Biomimetic strategies, by contrast, demonstrate a stronger alignment with thermodynamic and envelope-based system optimisation.

Recent post-2020 biophilic design research has similarly identified the rapid expansion of nature-based spatial strategies, yet has also pointed to persistent methodological dispersion and domain fragmentation [1,2,5,8]. These studies emphasise natural light, materiality, and spatial configuration as contributors to indoor environmental quality (IEQ), yet often lack systematic cross-domain performance validation.

The CPMF reframes this landscape. Rather than treating bio-inspiration as an aesthetic or conceptual alignment with nature, it positions both biophilic and biomimetic strategies within a structured performance-operational logic. By linking design intent to biophysical mechanism and measurable comfort output, the framework shifts the discourse from symbolic nature-referencing toward quantifiable environmental activation.

The persistent dominance of visually oriented biophilic configurations indicates that aesthetic–natural alignment continues to overshadow measurable performance optimisation. The CPMF addresses this imbalance by repositioning bio-inspiration within a thermodynamic and performance-operational logic rather than a purely perceptual or symbolic framework.

### 5.2. Structural Asymmetries Across Comfort Domains

The domain-level distribution (Table 3) reveals that multi-domain studies constitute 62.8% of the dataset, followed by visual comfort (25.6%). Thermal comfort represents only 10.3% as a primary domain, IAQ 1.3%, and acoustic performance remains entirely absent.

Although the prevalence of multi-domain studies may suggest integrative modelling, closer inspection indicates that many combine visual and thermal variables rather than comprehensively integrating acoustic and IAQ performance. This structural imbalance parallels broader IEQ discourse in recent sustainability literature, where integrated environmental performance frameworks are advocated but rarely operationalised across all comfort domains [16,17,23,35,36].

The absence of acoustic bio-inspired optimisation is particularly striking. This gap is theoretically paradoxical, given that several biological systems (e.g., porous natural fibres, avian feather structures, and hierarchical shell geometries) inherently demonstrate sound absorption and diffusion properties, yet remain under-translated into interior acoustic modelling. This under-translation highlights a missed opportunity for mechanism-driven acoustic optimisation within interior-scale biomimetic systems. Contemporary acoustic comfort research has advanced rapidly in the fields of environmental psychology and building physics; however, bio-inspired modelling has yet to meaningfully engage with this domain. This suggests a significant research frontier, particularly for biomimetic porous geometries and bio-based fibrous materials capable of sound absorption. This bottleneck appears to stem less from the absence of relevant biological analogues than from translational and methodological constraints in interior acoustic research. In contrast to thermal and daylight-oriented biomimetic studies—where simulation environments, façade-control logics, and performance metrics are already well established—acoustic biomimetic optimisation requires reliable coupling between material morphology, frequency-dependent sound behaviour, and interior performance indicators such as absorption, diffusion, or reverberation control. As a result, the current gap is better understood not as a lack of bio-inspired precedent, but as a lag in mechanism translation, performance operationalisation, and application-ready integration within interior acoustic modelling.

Similarly, IAQ-focused bio-inspired strategies remain predominantly plant-based and biophilic, with limited biomimetic system-level integration. This highlights an underdeveloped intersection between pollutant adsorption modelling and bio-inspired airflow regulation systems.

### 5.3. Empirical Maturity and Evidence Stratification

The evidence strength distribution (Table 6) reveals that only 10.3% of studies achieve Level A empirical validation, while 50% remain review-based or conceptually structured. Simulation-based studies account for 17.9%, reflecting a strong modelling orientation within biomimetic research.

Recent post-2020 studies on biomimetic adaptive façades and climate-responsive envelope systems demonstrate a strong reliance on computational modelling and simulation-based performance evaluation (Table 6). However, simulation dominance does not necessarily equate to validated interior deployment. Conversely, biophilic research frequently emphasises restorative perception and visual exposure while comparatively under-reporting measurable environmental performance outputs.

By embedding evidence strength as an explicit analytical layer, the CPMF enables differentiation between speculative bio-inspiration and performance-validated strategies.

### 5.4. Translational Feasibility and Implementation Constraints

A major theoretical advancement of the CPMF lies in the introduction of the implementation constraint layer. Contemporary sustainability discourse increasingly recognises that performance optimisation alone does not guarantee feasibility. Maintenance demand, lifecycle cost, scalability, and regulatory compatibility shape the real-world viability of interior environmental strategies.

For instance, plant-based IAQ systems may demonstrate measurable pollutant reduction while introducing maintenance complexity and moisture risks. Similarly, adaptive biomimetic façades may optimise thermal performance but increase cost intensity and operational complexity.

This translational dimension aligns with performance-oriented standards such as WELL and contemporary IEQ certification systems, where measurable environmental parameters are directly linked to occupant health and post-occupancy evaluation frameworks. By integrating feasibility constraints into the analytical chain, the CPMF extends beyond environmental modelling and engages with deployability realism.

The integration of evidence strength within the CPMF aligns bio-inspired interior research with evidence-based design paradigms. By embedding empirical validation as an explicit evaluative layer, the framework challenges concept-driven design discourse and advances toward performance-informed environmental strategy.

### 5.5. Theoretical Advancement: From Stylistic Paradigm to Mechanistic Model

The principal theoretical contribution of this study lies in repositioning biomimetic interior design from a stylistic or symbolic paradigm toward a mechanistic, evidence-aware performance model.

The CPMF formalises a biomimetic mechanism transfer pipeline linking biological logic to physical process activation, measurable IEQ outputs, empirical validation tiers, and implementation feasibility. These layers comprise design strategy, bio-logic mechanism, physical process activation, comfort output parameter, evidence strength, and implementation constraint.

Design strategies operate through bio-logic mechanisms that activate measurable physical processes within the built environment. These processes manifest as quantifiable comfort outputs, whose reliability is conditioned by empirical validation. Implementation constraints serve as a final evaluative layer, mediating between theoretical performance potential and practical applicability.

In doing so, the framework transforms fragmented literature clusters into a structured comparative system and provides a replicable template for future performance-oriented bio-inspired interior research.

From a sustainability epistemology perspective, the findings suggest a shift from symbolic ecological alignment toward operational environmental accountability, where environmental legitimacy increasingly depends on measurable performance claims and their evidential robustness. This shift parallels broader building performance and sustainability assessment debates that distinguish compliance-oriented and legitimacy-oriented accountability logics, and reflects ongoing debates on the divergence between modelling performance and empirical verification. At the indoor environmental scale, the persistent heterogeneity of IEQ models and indicators further reinforces the need for structured, transparent performance operationalisation across comfort domains [15,16,17].

## 6. Conclusions

This study advances the discourse on biomimetic interior design by repositioning it from a stylistic or symbolic paradigm toward a structured, performance-operational framework. Through systematic mapping and cross-domain comparative analysis of 78 peer-reviewed studies, the research demonstrates that contemporary bio-inspired interior research remains structurally differentiated rather than holistically integrated across environmental comfort domains.

The findings reveal three critical asymmetries. First, biophilic strategies dominate the literature and are primarily concentrated in visually oriented and multi-domain configurations. Second, biomimetic approaches exhibit stronger alignment with thermodynamic and envelope-based system optimisation but remain comparatively underrepresented in interior-scale applications. Third, acoustic performance remains entirely absent as a primary domain within bio-inspired interior research, indicating a significant and underexplored research frontier.

By integrating design strategy, bio-logic mechanism, physical process activation, comfort output parameter, evidence strength, and implementation constraints into a unified analytical structure, the Biomimetic Mechanism Transfer Mapping Framework (CPMF) transforms fragmented literature clusters into a mechanistic and implementation-aware model. The inclusion of evidence-tier stratification enables differentiation between concept-driven bio-inspiration and empirically validated performance strategies, while the implementation layer introduces translational feasibility as a critical evaluative dimension. In this sense, the feasibility layer is not a secondary addition to the CPMF but the point at which biomimetic transfer either becomes operational or fails to move beyond analogy. In current bio-inspired interior research, translational breakdown appears to occur most often at the interface between biological logic and implementation, where promising mechanisms encounter constraints of maintenance, controllability, cost, durability, and regulatory compatibility.

By introducing translational feasibility as a critical evaluative dimension, the framework extends beyond descriptive performance mapping. This study contributes to the field in five principal ways:

- It provides, to the author’s knowledge, one of the first structured mappings of biomimetic mechanism transfer across interior environmental comfort domains, using biophilic strategies as a comparative reference condition.
- It quantitatively identifies domain-level asymmetries, particularly the absence of acoustic bio-inspired optimisation.
- It embeds empirical validation tiers into comfort-domain mapping, revealing methodological fragmentation across research clusters.
- It introduces an implementation-constraint layer that links environmental performance to feasibility and real-world deployment.
- It proposes the CPMF as a replicable analytical template capable of guiding future performance-oriented bio-inspired interior research.

Collectively, these contributions reposition bio-inspired interior design from symbolic ecological alignment toward measurable environmental accountability.

Notwithstanding these contributions, several limitations should be acknowledged. The dataset was restricted to publications indexed in Scopus and Web of Science within a defined temporal window, which may exclude emerging research published outside these databases. The classification of primary comfort domains necessarily simplifies studies addressing multiple environmental parameters simultaneously. Evidence strength categorisation, while structured, reflects reporting quality rather than experimental superiority. In addition, the present dataset did not support a formal intervention scale × evidence strength contingency analysis; therefore, scale-related robustness comparisons remain descriptive. Furthermore, implementation constraints were analytically inferred rather than empirically measured, indicating the need for applied case-based validation.

Building upon these limitations, future research should prioritise three strategic directions. First, targeted investigation of acoustic bio-inspired modelling is urgently needed, particularly in relation to porous biomimetic geometries and bio-based absorptive materials. Second, empirical validation of biomimetic envelope strategies at interior scale is required to bridge the gap between simulation dominance and real-world deployment. Third, integrated IEQ modelling frameworks capable of simultaneously operationalising thermal, visual, acoustic, and IAQ performance should be developed to overcome persistent domain fragmentation. Longitudinal post-occupancy evaluation studies, coupled with lifecycle cost modelling and regulatory alignment analysis, would further strengthen translational feasibility assessment and support the evolution of bio-inspired interior research toward fully operational performance paradigms.

## Figures and Tables

**Table 1:** Distribution of intervention scale across included studies (*n* = 78).

Intervention Scale	Count	Percentage (%)
System scale (envelope/HVAC-integrated)	32	41.0
Component scale (façade modules/shading devices)	22	28.0
Material scale (bio-based/porous materials)	15	19.0
Spatial configuration scale	9	12.0
Total	78	100

**Table 2:** Distribution of design approaches across indoor comfort domains (*n* = 78).

Approach	Thermal	IAQ	Visual	Acoustic	Multi-Domain	Total
Biophilic	3 (5.4%)	0 (0.0%)	15 (26.8%)	0 (0.0%)	38 (67.9%)	56
Biomimetic	5 (26.3%)	1 (5.3%)	3 (15.8%)	0 (0.0%)	10 (52.6%)	19
Hybrid	0 (0.0%)	0 (0.0%)	2 (66.7%)	0 (0.0%)	1 (33.3%)	3
Total	8	1	20	0	49	78

**Table 3:** Distribution across primary comfort domains (*n* = 78).

Comfort Domain	Count	Percentage (%)
Multi-domain	49	62.8
Visual	20	25.6
Thermal	8	10.3
IAQ	1	1.3
Acoustic	0	0.0
Total	78	100

**Table 4:** Overall distribution of design approaches (*n* = 78).

Approach	Count	Percentage (%)
Biophilic	56	71.8
Biomimetic	19	24.4
Hybrid	3	3.8
Total	78	100

**Table 5:** Evidence type distribution (*n* = 78).

Evidence Type	Count	Percentage (%)
Review	21	26.9
Experimental	18	23.1
Simulation	14	17.9
Mixed	7	9.0
Unspecified	18	23.1
Total	78	100

**Table 6:** Evidence strength distribution (*n* = 78).

Evidence Strength	Count	Percentage (%)
A	8	10.3
B	17	21.8
C	14	17.9
D	39	50.0
Total	78	100

**Table 7:** Cross-tabulation of intervention type by evidence strength (A–D) in the final screened corpus (*n* = 78).

Intervention Type	A	B	C	D	Total
Acoustic treatment	1	0	0	1	2
Façade/envelope system	4	4	6	6	20
HVAC/ventilation system	0	4	1	3	8
Indoor vegetation system	1	2	2	5	10
Lighting/daylighting strategy	2	5	3	15	25
Unspecified	0	2	2	9	13
Total	8	17	14	39	78

Note: “Unspecified” indicates that the intervention type was not explicitly stated in the source paper and could not be reliably inferred from the available description. A distinction should be maintained between intervention type and primary comfort domain. The presence of studies coded as “acoustic treatment” in Table 7 does not indicate the existence of studies classified with acoustic comfort as the primary domain in Table 3; rather, these cases incorporated acoustic measures within broader or multi-domain intervention contexts.

**Table 8:** Cross-domain mapping of biomimetic mechanisms, environmental performance intersections, literature density, and evidence strength across the final screened corpus (*n* = 78).

Biophysical Mechanism	Thermal	IAQ	Visual	Acoustic	Multi-Domain
Evapotranspiration (Plant-based systems)	Microclimate cooling	VOC/CO_2_ reduction	Biophilic daylight synergy	No primary studies identified	Thermal–IAQ coupling
Adaptive Shading (Biomimetic facade)	Solar gain modulation	No primary studies identified	Daylight regulation	No primary studies identified	Thermal–Visual integration
Thermal Mass Modulation	Operative temperature stabilisation	Humidity buffering	No primary studies identified	No primary studies identified	Thermal–IAQ buffering
Porous/Bio-inspired Surfaces	No primary studies identified	Air filtration enhancement	Light diffusion	Potential sound absorption	IAQ–Visual–Acoustic nexus
Dynamic Envelope Systems	Cooling/heating demand reduction	Ventilation control	Glare mitigation	No primary studies identified	System-scale performance integration
Literature Density (*n* = 78)	Low (10.3%)	Very Low (1.3%)	Moderate (25.6%)	Absent (0%)	High (62.8%)
Implementation Constraints: Maintenance | Cost | Scalability | Code Compliance					
Evidence Strength Classification (*n* = 78): A—Empirical measurement with statistical validation (10.3%) B—Empirical measurement without detailed statistical reporting (21.8%) C—Simulation-based performance evaluation (17.9%) D—Review-based or conceptual analysis (50.0%)					

Note: Entries labelled “No primary studies identified” and the literature-density row are directly derived from the screened corpus. Mechanism–domain intersections such as “potential sound absorption,” “thermal–IAQ coupling,” or “system-scale performance integration” represent interpretive synthesis from the reviewed literature and are presented as analytical propositions rather than one-to-one empirical counts.

## Data Availability

The dataset supporting the findings of this study consists of peer-reviewed publications indexed in Scopus and Web of Science between 2020 and 2025. The coded analytical dataset generated during the systematic mapping process is available from the corresponding author upon reasonable request.

## Supplementary Materials

The following supporting information can be downloaded at: https://www.mdpi.com/article/10.3390/biomimetics11040225/s1, PRISMA-ScR checklist.
