# Supplementary material for: Biomimetic Mechanism Transfer in Interior Environmental Comfort: A Systematic Mapping and Evidence-Stratified Framework
Source: Biomimetics (Basel). 2026 Mar 25;11(4):225. doi: 10.3390/biomimetics11040225 (PMC13113413; doi:10.3390/biomimetics11040225)
Supplement: Supplementary file 1 [file biomimetics-11-00225-s001.zip › biomimetics-4208595-supplementary.pdf]

Preferred Reporting Items for Systematic reviews and Meta-Analyses extension for Scoping Reviews (PRISMA-ScR) Checklist

SECTION	ITEM	PRISMA-ScR CHECKLIST ITEM	REPORTED ON PAGE #
**TITLE**			
Title	1	Identify the report as a scoping review.	1
**ABSTRACT**			
Structured summary	2	Provide a structured summary that includes (as applicable): background, objectives, eligibility criteria, sources of evidence, charting methods, results, and conclusions that relate to the review questions and objectives.	1
**INTRODUCTION**			
Rationale	3	Describe the rationale for the review in the context of what is already known. Explain why the review questions/objectives lend themselves to a scoping review approach.	2-3
Objectives	4	Provide an explicit statement of the questions and objectives being addressed with reference to their key elements (e.g., population or participants, concepts, and context) or other relevant key elements used to conceptualize the review questions and/or objectives.	3
**METHODS**			
Protocol and registration	5	Indicate whether a review protocol exists; state if and where it can be accessed (e.g., a Web address); and if available, provide registration information, including the registration number.	No review protocol was prospectively registered. Therefore, no registration number is available.
Eligibility criteria	6	Specify characteristics of the sources of evidence used as eligibility criteria (e.g., years considered, language, and publication status), and provide a rationale.	6–7. Studies were eligible if they were peer-reviewed journal articles or review articles published between 2020 and 2025 and indexed in Scopus or Web of Science. Included studies explicitly addressed biomimetic mechanism transfer, biophilic strategies (as a comparative reference), or hybrid biologically informed design approaches at building or interior scale, and either reported measurable indoor environmental performance outcomes or explicitly analysed performance-relevant indoor environmental parameters, mechanisms, or evaluation frameworks, even when no original

St.Michael's
Inspired Care.
Inspiring Science.

SECTION	ITEM	PRISMA-ScR CHECKLIST ITEM	REPORTED ON PAGE #
			empirical or simulation-based outputs were generated. Studies had to address at least one predefined comfort domain (thermal, IAQ, visual, or acoustic). Conference proceedings, book chapters, editorials, and non-peer-reviewed materials were excluded to ensure methodological robustness and comparability.
Information sources*	7	Describe all information sources in the search (e.g., databases with dates of coverage and contact with authors to identify additional sources), as well as the date the most recent search was executed.	6. The information sources used for the search were Scopus and Web of Science Core Collection. The database search was conducted in January 2026.
Search	8	Present the full electronic search strategy for at least 1 database, including any limits used, such that it could be repeated.	6. The core search string used across databases was: (“biophilic design” OR biophilia OR biomimicry OR “biomimetic design”) AND (“thermal comfort” OR “acoustic comfort” OR “indoor air quality” OR “visual comfort” OR lighting) AND (“interior design” OR “building design” OR “built environment”). Searches were limited to Title–Abstract–Keywords fields in Scopus and Topic fields in Web of Science and restricted to the publication period 2020–2025.
Selection of sources of evidence†	9	State the process for selecting sources of evidence (i.e., screening and eligibility) included in the scoping review.	6–7. Records were identified through database searches in Scopus (n = 53) and Web of Science (n = 47), yielding 100 records. After removing 12 duplicate records using DOI matching, 88 records remained. Title–abstract screening was conducted according to predefined eligibility criteria, resulting in the exclusion of 10 records. A total of 78 studies were included in the final qualitative mapping analysis, as illustrated in the PRISMA flow diagram.
Data charting process‡	10	Describe the methods of charting data from the included sources of evidence (e.g., calibrated forms or forms that have been tested by the team before their use, and whether data charting was done independently or in duplicate) and any processes for obtaining and confirming data from investigators.	7–8. A structured coding matrix was developed to enable systematic comparison across the included studies. Each study was coded according to predefined analytical categories. To enhance methodological robustness, a subset of the dataset (15% of the included studies) was re-examined by the author after the initial coding

SECTION	ITEM	PRISMA-ScR CHECKLIST ITEM	REPORTED ON PAGE #
			phase to assess coding stability and categorical consistency. Ambiguous cases were revisited through iterative comparison with the predefined coding rules.
Data items	11	List and define all variables for which data were sought and any assumptions and simplifications made.	6–7. The charted variables included design approach (biophilic, biomimetic, hybrid), primary comfort domain (thermal, IAQ, visual, acoustic, or multi-domain), intervention type, evidence type (experimental, simulation, mixed, review), reported performance metrics (e.g., PMV, CO_2 concentration, illuminance, RT60), and evidence strength classification (A–D).
Critical appraisal of individual sources of evidence§	12	If done, provide a rationale for conducting a critical appraisal of included sources of evidence; describe the methods used and how this information was used in any data synthesis (if appropriate).	6–7. A formal risk-of-bias or critical appraisal assessment was not conducted. Instead, empirical robustness was evaluated through an evidence strength classification (A–D), distinguishing between statistically validated empirical studies, empirical measurements without detailed statistical reporting, simulation-based studies, and conceptual or review-based analyses.
Synthesis of results	13	Describe the methods of handling and summarizing the data that were charted.	9. The charted data were synthesised through quantitative mapping of biomimetic mechanism transfer across indoor environmental comfort domains, cross-tabulation of intervention type and evidence type, and identification of underrepresented bio-inspired performance intersections. These analytical steps provided the empirical basis for the development of the Comparative Performance Mapping Framework (CPMF).
**RESULTS**			
Selection of sources of evidence	14	Give numbers of sources of evidence screened, assessed for eligibility, and included in the review, with reasons for exclusions at each stage, ideally using a flow diagram.	6–7. The database search identified 100 records (Scopus n = 53; Web of Science n = 47). After removing 12 duplicates, 88 records remained. Title–abstract screening resulted in the exclusion of 10 records, and 78 studies were included in the final qualitative mapping analysis (Figure 1). The flow diagram reports the aggregate number excluded at the

St. Michael's
Inspired Care.
Inspiring Science.

SECTION	ITEM	PRISMA-ScR CHECKLIST ITEM	REPORTED ON PAGE #
			title–abstract screening stage; the exclusion criteria applied at this stage are specified in Section 3.4 (E1: urban/outdoor context without indoor environmental relevance; E2: psychological perception without measurable environmental metrics; E3: purely conceptual without reference to measurable performance outcomes; E5: non-built-environment domains).
Characteristics of sources of evidence	15	For each source of evidence, present characteristics for which data were charted and provide the citations.	7–8. The characteristics of the included sources of evidence were charted using predefined analytical categories including design approach (biophilic, biomimetic, hybrid), comfort domain (thermal, IAQ, visual, acoustic, or multi-domain), intervention scale, evidence type, and reported performance metrics. The dataset included 78 peer-reviewed studies analysed through this structured coding framework.
Critical appraisal within sources of evidence	16	If done, present data on critical appraisal of included sources of evidence (see item 12).	13-14. No formal risk-of-bias appraisal was conducted. However, empirical robustness was examined through an evidence strength classification (A–D). The distribution of evidence strength across the included studies is presented in Table 6, distinguishing between statistically validated empirical studies, empirical measurements, simulation-based studies, and conceptual or review-based analyses.
Results of individual sources of evidence	17	For each included source of evidence, present the relevant data that were charted that relate to the review questions and objectives.	10–14. The results of the included studies were analysed according to predefined analytical categories including design approach and indoor comfort domain. The findings reveal a predominance of biophilic strategies (71.8%) compared with biomimetic (24.4%) and hybrid approaches (3.8%). Multi-domain environmental studies accounted for 62.8% of the dataset, followed by visual (25.6%) and thermal (10.3%) domains, while acoustic bio-inspired optimisation was absent.
Synthesis of results	18	Summarize and/or present the charting results as they relate to	14–17. The charted evidence was synthesised through comparative analysis of design approaches,

SECTION	ITEM	PRISMA-ScR CHECKLIST ITEM	REPORTED ON PAGE #
		the review questions and objectives.	comfort domains, and evidence strength levels. These analytical results were integrated into the Comparative Performance Mapping Framework (CPMF), which structures biomimetic mechanism transfer across biological logic, physical processes, environmental performance outputs, empirical robustness, and implementation feasibility.
**DISCUSSION**			
Summary of evidence	19	Summarize the main results (including an overview of concepts, themes, and types of evidence available), link to the review questions and objectives, and consider the relevance to key groups.	17–19. The review reveals structural asymmetries in bio-inspired interior research, with biophilic strategies dominating the literature and biomimetic strategies more strongly associated with thermodynamic and system-level optimisation. The analysis highlights significant gaps, particularly the absence of acoustic bio-inspired optimisation and the uneven empirical validation across comfort domains. These findings support the need for an integrated analytical framework linking biological mechanisms to measurable indoor environmental performance outcomes.
Limitations	20	Discuss the limitations of the scoping review process.	20. Several limitations should be acknowledged. The dataset was restricted to publications indexed in Scopus and Web of Science within the 2020–2025 time frame, which may exclude relevant studies published elsewhere. The classification of primary comfort domains simplifies studies addressing multiple environmental parameters simultaneously. In addition, implementation constraints were analytically inferred rather than empirically measured.
Conclusions	21	Provide a general interpretation of the results with respect to the review questions and objectives, as well as potential implications and/or next steps.	20–21. The study concludes that biomimetic interior design should be repositioned from a symbolic or stylistic paradigm toward a performance-oriented framework linking biological mechanisms to measurable indoor environmental outcomes. The proposed Comparative Performance Mapping Framework (CPMF) provides a structured model for analysing

SECTION	ITEM	PRISMA-ScR CHECKLIST ITEM	REPORTED ON PAGE #
			biomimetic mechanism transfer and highlights key research gaps, particularly the absence of acoustic bio-inspired optimisation and the need for stronger empirical validation across indoor environmental quality domains.
**FUNDING**			
Funding	22	Describe sources of funding for the included sources of evidence, as well as sources of funding for the scoping review. Describe the role of the funders of the scoping review.	21. This research received no external funding.

JBI = Joanna Briggs Institute; PRISMA-ScR = Preferred Reporting Items for Systematic reviews and Meta-Analyses extension for Scoping Reviews.

* Where *sources of evidence* (see second footnote) are compiled from, such as bibliographic databases, social media platforms, and Web sites.

† A more inclusive/heterogeneous term used to account for the different types of evidence or data sources (e.g., quantitative and/or qualitative research, expert opinion, and policy documents) that may be eligible in a scoping review as opposed to only studies. This is not to be confused with *information sources* (see first footnote).

‡ The frameworks by Arksey and O'Malley (6) and Levac and colleagues (7) and the JBI guidance (4, 5) refer to the process of data extraction in a scoping review as data charting.

§ The process of systematically examining research evidence to assess its validity, results, and relevance before using it to inform a decision. This term is used for items 12 and 19 instead of "risk of bias" (which is more applicable to systematic reviews of interventions) to include and acknowledge the various sources of evidence that may be used in a scoping review (e.g., quantitative and/or qualitative research, expert opinion, and policy document).

From: Tricco AC, Lillie E, Zarin W, O'Brien KK, Colquhoun H, Levac D, et al. PRISMA Extension for Scoping Reviews (PRISMAScR): Checklist and Explanation. Ann Intern Med. 2018;169:467–473. doi: 10.7326/M18-0850.
